# The triglyceride-to-high-density lipoprotein cholesterol ratio is associated with an increased risk of peripartum cardiomyopathy

**DOI:** 10.3389/fendo.2024.1447791

**Published:** 2024-11-19

**Authors:** Naiyi Chen, Jie Xiao, Yijie Luo, Kang Fu, Ziru Sun, Xinyu Zhang, Yanan Liu, Huixia Lu, Xiaoping Ji

**Affiliations:** ^1^ The Key Laboratory of Cardiovascular Remodeling and Function Research, The State and Shandong Province Joint Key Laboratory of Translational Cardiovascular Medicine, Department of Cardiology, Cheeloo College of Medicine, Qilu Hospital, Chinese Ministry of Education, Chinese National Health Commission and Chinese Academy of Medical Sciences, Shandong University, Jinan, China; ^2^ Department of Critical Care Medicine, Qilu Hospital of Shandong University, Jinan, China; ^3^ Department of Obstetrics and Gynecology, The People’s Hospital of Qihe County, Dezhou, Shandong, China

**Keywords:** peripartum cardiomyopathy, metabolism, blood lipids, risk, TG/HDL-C ratio

## Abstract

**Background:**

Peripartum cardiomyopathy (PPCM) is a form of heart failure that severely affects women during the late stages of pregnancy and in the postpartum period. Currently, the diagnosis of PPCM is not fully understood and is likely multifactorial. Abnormal lipid metabolism plays an important role in the onset of cardiovascular diseases, especially in patients with heart failure. Moreover, animal experiments have confirmed a possible association between abnormal lipid metabolism and PPCM onset. However, clinical evidence is currently lacking, and reliable and effective indicators for predicting the onset of PPCM are insufficient. The ratio of triglyceride to high-density lipoprotein cholesterol (TG/HDL-C) is a novel metabolic marker that is associated with the risk of coronary heart disease. However, the relationship between the TG/HDL-C ratio and the risk of PPCM in parturients remains unknown. Therefore, this study aimed to explore the role of the TG/HDL-C ratio in predicting the risk of PPCM.

**Methods:**

This study included 600 parturients hospitalized at Qilu Hospital of Shandong University between January 2010 and August 2023 (150 patients with PPCM and 450 healthy parturients). The TG/HDL-C ratio was calculated as triglyceride levels (mmol/L)/high-density lipoprotein cholesterol levels (mmol/L). The relationship between the TG/HDL-C ratio and PPCM was analyzed using logistic regression analysis and receiver operating characteristic curve analysis.

**Results:**

Significant differences were observed in the TG/HDL-C ratio between patients with PPCM and healthy parturients. The TG/HDL-C ratio was higher in patients with PPCM than in healthy parturients (*p* < 0.001). Logistic regression analysis revealed that the TG/HDL-C ratio increased the risk of PPCM and had predictive accuracy for the onset of PPCM (odds ratio, 1.249; 95% confidence interval, 1.004–1.553; *p* = 0.046). Moreover, the TG/HDL-C ratio was positively correlated with the NT-proBNP levels at the onset of PPCM (*R* ^2^ = 0.081, *p* = 0.008).

**Conclusions:**

A higher TG/HDL-C ratio was significantly associated with the risk of PPCM.

## 1 Introduction

Peripartum cardiomyopathy (PPCM) is a form of heart failure (HF) that develops in previously healthy women during the late stages of pregnancy or in the months following delivery. It is characterized by idiopathic cardiomyopathy with prominent left ventricular systolic dysfunction (1).

PPCM is defined by a left ventricular ejection fraction (LVEF) of <45%, typically accompanied by dilation of the left ventricle (1). Some patients with LVEF >45% who exhibit the typical features of PPCM may also be diagnosed with PPCM (1). The incidence of PPCM varies globally and racially, as previous studies have shown, ranging from 1:200 pregnancies in Nigeria (2) to 1:20,000 in Japan (3). This is due to the subtle clinical manifestations in some patients with PPCM and variations in the diagnosis and treatment modalities across different regions. Thus, the real-world incidence of PPCM may be underestimated. As one of the leading causes of peripartum maternal mortality, PPCM not only has severe impacts on maternal daily life but also affects fetal growth and development.

The etiology of PPCM remains unclear. Its main risk factors include unhealthy nutrition, lack of physical activity, dyslipidemia, hyperglycemia, hypertension, preeclampsia, obesity, older age, race/nationality, thrombosis/smoking, renal dysfunction, and hereditary or familial hypercholesterolemia (4). Studies have shown that multiple births, a family history of cardiomyopathy, diabetes, and long-term use of tocolytic agents increase the risk of PPCM (5). A better understanding of the risk factors for PPCM can help identify high-risk individuals earlier, enabling its timely monitoring and treatment. Dyslipidemia is an important risk factor for cardiovascular diseases (6). Sphingomyelin (7), phosphatidylcholine, and triglycerides, as components of the cardiac lipid panel, are relevant to cardiomyocyte stress and coronary artery disease and comprise one prognostic score in the risk assessment of chronic HF, which is conducted using a combination of the cardiac lipid profile and NT-proBNP (8). Comprehensive lipidomic studies on patients with HF and their control group revealed a significant positive correlation between neuroamide, phosphatidylcholine, and ceramide levels and the risk of HF (9). Homocysteine (HCY) is a sulfhydryl-containing amino acid produced during the metabolism of methionine and cysteine (21). The increase in HCY levels is considered a risk factor for cardiovascular diseases. Another study revealed that elevated maternal HCY levels were associated with various perinatal complications and postpartum syndromes. However, whether the HCY levels are associated with PPCM remains unclear (10).

During pregnancy, the increasing demand for energy and nutrition results in increased food intake, which has various effects on lipid metabolism. In addition, lipid storage typically increases under the influence of pregnancy-related hormones. Some women also experience insulin resistance (11). Based on the aforementioned studies, we hypothesized that patients with PPCM might have lipid metabolic abnormalities.

The diagnosis of PPCM is exclusionary and requires the exclusion of HF resulting from other causes, with no definitive predictive markers currently available. The similarity between HF symptoms in patients with PPCM and normal pregnancy symptoms often leads to delayed diagnosis and treatment, underscoring the urgent need for reliable predictive indicators to aid in the early identification of high-risk PPCM individuals and prompt intervention and treatment. It is particularly important to explore sensitive, specific, simple, and feasible indicators based on existing routine prenatal monitoring. The ratio of triglyceride to high-density lipoprotein cholesterol (TG/HDL-C) is closely related to insulin resistance, which is the basis of the pathogenesis of metabolic syndrome. Related studies have proposed that the TG/HDL-C ratio can be used as an evaluation index for metabolic syndrome and have revealed that individuals with higher TG/HDL-C ratio are more likely to develop diabetes mellitus (12). Furthermore, the TG/HDL-C ratio is associated with a high risk of cerebrovascular disease and the prognosis of kidney diseases (13). Studies have also proposed that the TG/HDL-C ratio of patients with coronary heart disease is higher than that of healthy controls and that the TG/HDL-C ratio can help predict coronary heart disease (14). The TG/HDL-C ratio may be relevant to outcomes in patients with myocardial infarction (12). It is a composite index that is convenient to obtain, is simple to calculate, and can be easily derived from routine laboratory tests at a low cost without increasing the economic burden on patients or imposing additional trauma or risks. Existing research suggests that metabolic abnormalities may increase the risk of PPCM; however, no clear metabolic markers are available to screen for PPCM risk. Moreover, whether TG/HDL-C ratios are related to the development of PPCM remains unclear. Therefore, this study aimed to explore the role of the TG/HDL-C ratio in predicting the risk of PPCM.

## 2 Materials and methods

### 2.1 Study population

In this retrospective study, we obtained data from the medical record system of Qilu Hospital, Shandong University. We screened patients with PPCM hospitalized in the Departments of Obstetrics and Cardiology and the intensive care unit in Qilu Hospital, Shandong University, from January 2010 to August 2023. Furthermore, we recorded telephone follow-ups. The diagnostic criteria for PPCM were as follows: peripartum status, LVEF less than 45%, no identifiable cause of HF, and patients who underwent blood lipid tests and echocardiography on admission. The exclusion criteria were as follows: history of heart diseases, other identifiable causes of HF, severe infection or severe impairment of liver and kidney functions [ALT/AST > 5 times the normal upper limit; estimated glomerular filtration rate (eGFR) <30 mL/min/1.73 m^2^], no blood lipid test and echocardiography on admission, history of malignant tumors, and history of rheumatic and autoimmune diseases. Healthy parturients who delivered at Qilu Hospital in the same month were randomly selected from the hospital’s admission system as controls at a ratio of 1:3. The inclusion criteria for the control group were as follows: delivery at Qilu Hospital in the same month; availability of complete blood routine, coagulation, and biochemical examination results on admission; absence of HF during hospitalization; and no occurrence of obvious symptoms such as chest tightness or shortness of breath during telephone follow-ups for several months postpartum. Pregnant women who experienced HF during the peripartum period were excluded from the control group.

### 2.2 Data collection

Before sampling, we ensured that all study participants signed informed consent forms and understood the research purpose and methods. Data collected included the patient’s general condition, health status, reproductive history, vital signs, and laboratory tests at the time of admission. Moreover, the details regarding patients with PPCM also included data on echocardiography and N-terminal pro-brain natriuretic peptide (NT-proBNP) levels. Their general condition information included their names, ages, hospital numbers, contact information, and dates of admission and discharge. Details on their previous pregnancies included information on gravidity, delivery, gestational age at diagnosis, and multiple pregnancies. Laboratory tests at the time of admission were recorded, including fasting liver function (alanine aminotransferase and aspartate aminotransferase), kidney function (creatinine and eGFR), blood glucose, blood electrolytes (potassium), blood lipids (triglycerides, cholesterol, low-density lipoprotein cholesterol, and high-density lipoprotein cholesterol), and routine blood tests (leukocytes, monocytes, hemoglobin, and platelets). Blood samples were collected in a fasting state on the morning of the second day after admission between 6 and 7 a.m. to minimize the impact of physiological fluctuations on the results. The blood samples were drawn from the vein using sterile techniques, typically collecting 10–20 mL of blood. EDTA tubes were used for whole blood to be sent for a complete blood count, whereas centrifuge tubes were used for serum or plasma to be sent for liver and kidney function, electrolyte, and lipid tests. The samples were sent to the laboratory for analysis within 2 h of collection. Hematological analysis was used to assess the blood cells, and an automatic biochemical analysis instrument was used to determine the biochemical indices of the blood samples, such as triglycerides, cholesterol, and blood glucose. The TG/HDL-C index was calculated as the ratio of TG (mmol/L) to HDL-C (mmol/L). The vital echocardiographic indicators in the patients with PPCM included left atrial diameter, left ventricular diameter, right atrial diameter, right ventricular diameter, and LVEF.

### 2.3 Statistical analysis

Statistical analyses were performed on the baseline clinical data of the patients with PPCM and healthy control parturients. All data were subjected to the Kolmogorov–Smirnov normality test. Regarding the quantitative data, data that conformed to normal distribution are expressed as mean ± standard deviation, and those that did not conform are presented as median (first quartile, third quartile). Qualitative data are presented as rates or percentages. If the quantitative variables conformed to a normal distribution, the Student’s *t*-test was used to compare the two groups, and the Mann–Whitney *U* test or Wilcoxon test was used to compare the variables between groups that did not conform to the normal distribution.

A pairwise comparison between the TG/HDL-C ratio in patients with PPCM and the normal control group was performed using the non-parametric Wilcoxon signed-rank test. Qualitative variables were analyzed using the chi-square or Fisher’s exact test. The outcome variable was defined as the occurrence or non-occurrence of PPCM. Whether TG/HDL-C increased the risk of PPCM in perinatal women was evaluated using univariate logistic regression and multivariate logistic analyses. Based on previous studies on PPCM risk factors, comparisons of baseline data, and clinical observations, variables showing differences and those not showing differences but previously associated with PPCM risk were separately subjected to a single-factor logistic analysis for PPCM occurrence. Variables showing statistical significance in the single-factor logistic regression analysis and those not showing statistical significance but previously associated with PPCM risk were separately subjected to a single-factor logistic analysis for PPCM occurrence. This study aimed to clarify whether changes in these factors would increase or decrease the risk of PPCM.

Receiver operating characteristic (ROC) curve analysis was performed to investigate the predictive accuracy of the TG/HDL-C ratio for PPCM. The correlations between the TG/HDL-C ratio and HF indices at the presentation of HF, such as NT-proBNP, LVEF, and left ventricular end-diastolic diameter (LVEDD), were analyzed using Pearson’s correlation analysis. A two-tailed *p*-value < 0.05 was considered statistically significant. The data were analyzed using SPSS version 29.0, GraphPad Prism version 9, and R version 4.2.3.

## 3 Results

### 3.1 Baseline characteristics

**Figure 1:**
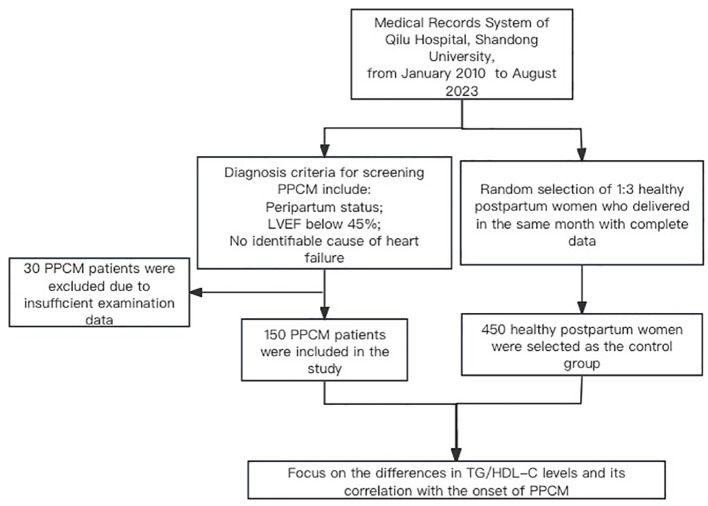
Study population. Peripartum cardiomyopathy, PPCM; triglyceride-to-high-density lipoprotein cholesterol ratio, TG/HDL-C.

From the medical records system of Qilu Hospital, 180 patients with PPCM who met the diagnostic criteria were screened. Among these, 30 patients with PPCM who lacked laboratory indicators and/or vital signs at admission were excluded. In total, 150 patients with PPCM were included in this study. Moreover, 450 healthy perinatal women without any PPCM episodes were randomly selected as the control group (**Figure 1**). Differences in the baseline data between the two groups are presented in **Table 1**. In this study, the rate of multiple pregnancies was significantly higher in the PPCM group than in the control group (*p* < 0.001). There were no statistically significant differences between the two groups in terms of age (*p* = 0.090) or multiparity (*p* = 0.833). The systolic blood pressure (SBP), diastolic blood pressure, heart rate, and incidence rate of sinus tachycardia between the two groups were higher in patients with PPCM (*p* < 0.001) (**Table 1**).

**Table 1:** Comparison of baseline characteristics between the two groups.

Variables	PPCM group *N* = 150	Control group *N* = 450	*p*
General information			
Age (years)	29.2 ± 6.0	30.8 ± 4.3	0.090
Multipara (%)	94 (62.67)	274 (60.89)	0.833
Multiple pregnancies (%)	17 (11.33)	13 (2.89)	<0.001
SBP (mm Hg)	122 ± 30	120 ± 12	0.007
DSP (mmHg)	81 ± 21	74 ± 9	<0.001
HR (bpm)	94 ± 21	93 ± 14	<0.001
Sinus tachycardia (%)	79 (52.67)	148 (32.89)	<0.001
BMI (kg/m^2^)	27.45 ± 5.64	27.60 ± 3.41	0.281
Complete blood count			
WBC (×10^9^/L)	8.13 ± 3.65	8.51 ± 2.34	0.271
NEU (%)	65.61 ± 11.30	73.03 ± 7.25	<0.001
LYM (%)	26.26 ± 10.20	19.88 ± 6.06	<0.001
MON (%)	6.27 ± 1.91	6.01 ± 1.93	0.348
HGB (g/L)	118 ± 20	116 ± 13	0.251
Anemia (%)	81 (54.00)	211 (46.89)	0.098
PLT (×10^9^/L)	248 ± 87	213 ± 56	<0.001
Coagulation series			
DD (μg/mL)	1.36 ± 1.55	1.23 ± 1.59	0.702
APTT-S (s)	31.55 ± 4.77	28.93 ± 3.74	<0.001
PT-S (s)	13.99 ± 9.34	11.04 ± 1.41	<0.001
FIB (g/L)	3.31 ± 0.98	4.40 ± 1.54	<0.001
Liver function			
ALT (U/L)	17.00 (10.00, 33.00)	9.00 (7.00, 12.50)	<0.001
AST (U/L)	24.00 (19.00, 34.00)	16.00 (13.00, 19.00)	<0.001
ALB (mmol/L)	34.80 ± 6.90	35.30 ± 5.30	<0.001
Hypoalbuminemia (%)	43 (28.67)	20 (4.44)	0.002
Kidney function			
Cr (μmol/L)	61.00 (51.50, 75.00)	49.00 (44.00, 55.00)	<0.001
eGFR (mL/min)	103.94 ± 16.23	111.38 ± 5.65	<0.001
UA (μmol/L)	428 ± 166	276 ± 69	<0.001
Blood lipids			
LDL-C (mmol/L)	2.86 ± 1.12	2.94 ± 0.87	0.591
HDL-C (mmol/L)	1.17 ± 0.52	1.94 ± 0.40	<0.001
TG (mmol/L)	2.13 ± 1.13	3.02 ± 1.23	<0.001
TC (mmol/L)	4.73 ± 1.70	5.91 ± 1.13	<0.001
**TG/HDL-C**	1.71 (1.44, 2.91)	1.47 (1.10, 1.99)	<0.001
Glu (mmol/L)	4.52 ± 1.20	4.15 ± 1.04	0.014
K (mmol/L)	4.22 ± 0.55	4.14 ± 0.38	0.812

Data presented as mean ± standard deviation, median (first quartile, third quartile) or number (percentage).

PPCM, Peripartum cardiomyopathy; BMI, body mass index; SBP, systolic blood pressure; HR, heart rate; WBC, white blood cell count; NEU%, neutrophil percentage; LYM%, lymphocyte percentage; MONO%, monocyte percentage; HGB, hemoglobin; PLT, platelet; DD, D-dimer; APTT, activated partial thromboplastin time; PT, prothrombin time; FIB, fibrinogen; ALT, alanine aminotransferase; AST, aspartate aminotransferase; ALB, albumin; Cr, creatinine; eGFR, estimated glomerular filtration rate; UA, uric acid; LDL-C, low-density lipoprotein cholesterol; HDL-C, high-density lipoprotein cholesterol; TG, triglycerides; TC, total cholesterol; FBG, fasting plasma glucose; K, potassium ion; TG/HDL-C, triglyceride-to-high-density lipoprotein cholesterol ratio.

**Figure 2:**
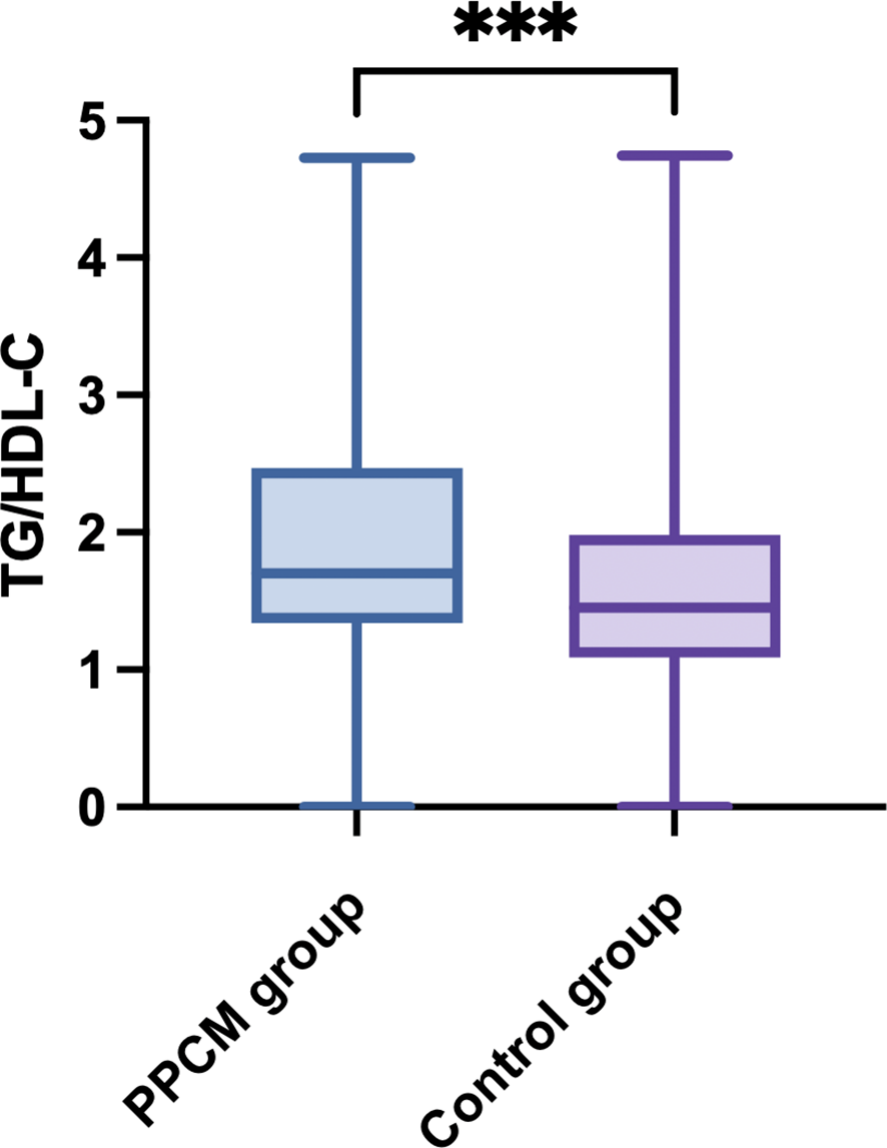
Comparison of TG/HDL-C between the PPCM group and the control group. Peripartum cardiomyopathy, PPCM; triglyceride-to-high-density lipoprotein cholesterol ratio, TG/HDL-C. ***p < 0.001.

There was no statistical difference between the two groups in terms of low-density lipoprotein cholesterol (LDL-C) levels (*p* = 0.591). However, the high-density lipoprotein cholesterol (HDL-C), triglyceride (TG), and total cholesterol (TC) levels were significantly lower in patients with PPCM than in the healthy controls (*p* < 0.001), while the FBG levels were significantly higher in those with PPCM (*p* = 0.014). Moreover, the TG/HDL-C ratio in patients with PPCM was significantly higher than that in the healthy controls (*p* < 0.001) (**Figure 2**).

Approximately 61.33% of the patients with PPCM had severe symptoms (NYHA Classes III and IV) (**Table 2**). The echocardiography of the patients with PPCM revealed that the mean LVEF was 32.3% ± 10.5%, the mean LVEDD was 58.1 ± 7.4 mm, and the mean right ventricular end-diastolic diameter was 23.1 ± 4.7 mm. Approximately 76.8% of the patients with PPCM had pulmonary arterial hypertension (**Table 2**).

**Table 2:** Baseline characteristics of PPCM group.

PPCM group *N* = 150		
NYHA (%)	II	58 (38.67)
	III	62 (41.33)
	IV	30 (20.00)
Comorbidities (%)	Gestational hypertension	51 (34.00)
	Gestational diabetes	3 (2.00)
	Chronic hypertension	3 (2.00)
Echocardiography	LA-aq (mm)	40.1 ± 5.3
	LVEDd (mm)	58.1 ± 7.4
	RVEDd (mm)	23.1 ± 4.7
	IVS (mm)	9.2 ± 1.8
	PASP (mmHg)	39.0 ± 13.6
	E/e’	15.6 ± 5.6
	LVEF (%)	32.3 ± 10.5
NT-proBNP (ng/mL)		2,505.0 (903.0, 6,628.0)
CRP (mg/L)		21.85 (13.31, 34.08)

PPCM, Peripartum cardiomyopathy; NYHA, New York Heart Association; LA-aq, left atrial anteroposterior diameter; LVEDd, left ventricular end diastolic diameter; RVEDd, right ventricular end diastolic diameter; IVS, interventricular septum; PASP, pulmonary artery systolic pressure; E/e’, the ratio of the early diastolic peak velocity of blood flow to early diastolic TDI annular velocities; LVEF, left ventricular ejection fraction; NT-proBNP, N-terminal B-type natriuretic peptide; CRP, C-reactive protein.

### 3.2 Association between TG/HDL-C and PPCM

Based on previous research on the risk factors for PPCM, comparison of the baseline data, and clinical observations, we selected variables including age, parity status, multiple pregnancies, SBP, BMI, HGB, ALB, and TG/HDL-C ratio for the single-factor logistic analysis of PPCM incidence. This study aimed to determine whether changes in these factors could increase or decrease the risk of PPCM. The results of the single-factor logistic analysis indicated that a higher rate of multiple pregnancies [odds ratio (OR), 4.297; 95% confidence interval (CI), 2.034–9.076; *p* < 0.001], the SBP (OR, 1.028; 95% CI, 1.015–1.041; *p* < 0.001), and the TG/HDL-C ratio (OR, 1.362; 95% CI, 1.117–1.660; *p* < 0.001) increased the risk of PPCM, while higher ALB levels (OR, 0.938; 95% CI, 0.904–0.973; *p* < 0.001) decreased the risk of PPCM.

Variables with statistical significance in the univariable logistic regression analysis and those that were not statistically significant but were related to the PPCM incidence risk in previous studies were included in the multivariate logistic regression analysis of PPCM incidence (**Table 3**). Variables such as age, parity status, multiple pregnancies, SBP, ALB, and TG/HDL-C ratio were included in the multivariate logistic regression model. The results of the multivariate regression analysis indicated that multiple pregnancies (OR, 6.824; 95% CI, 2.041–22.815; *p* = 0.046) and the TG/HDL-C ratio (OR, 1.249; 95% CI, 1.004–1.553; *p* = 0.046) increased the risk of PPCM in puerperal women (**Table 4**).

**Table 3:** Univariate regression analysis on the above variables and the incidence of PPCM.

Variables	OR	95% CI	*p*
Age	0.965	0.926–1.004	0.085
Multipara	0.965	0.814–1.145	0.682
Multiple pregnancies	4.297	2.034–9.076	<0.001
SBP	1.028	1.015–1.041	<0.001
BMI	1.029	0.972–1.089	0.327
HGB	0.993	0.981–1.006	0.272
ALB	0.938	0.904–0.973	<0.001
TG/HDL-C	1.362	1.117–1.660	0.002

PPCM, Peripartum cardiomyopathy; OR, odds ratio; CI, confidence interval; SBP, systolic blood pressure; BMI, body mass index; HGB, hemoglobin; ALB, albumin; TG/HDL-C, triglyceride-to-high-density lipoprotein cholesterol ratio.

**Table 4:** Multivariate regression analysis on the above variables and the incidence of PPCM.

	OR	95% CI	*p*
Age	0.964	0.908–1.024	0.373
Multiple pregnancies	6.824	2.041–22.815	0.002
SBP	1.018	1.002–1.034	0.027
ALB	0.974	0.930–1.020	0.256
TG/HDL-C	1.249	1.004–1.553	0.046

PPCM, Peripartum cardiomyopathy; OR, odds ratio; CI, confidence interval; SBP, systolic blood pressure; ALB, albumin; TG/HDL-C, triglyceride-to-high-density lipoprotein cholesterol ratio.

**Figure 3:**
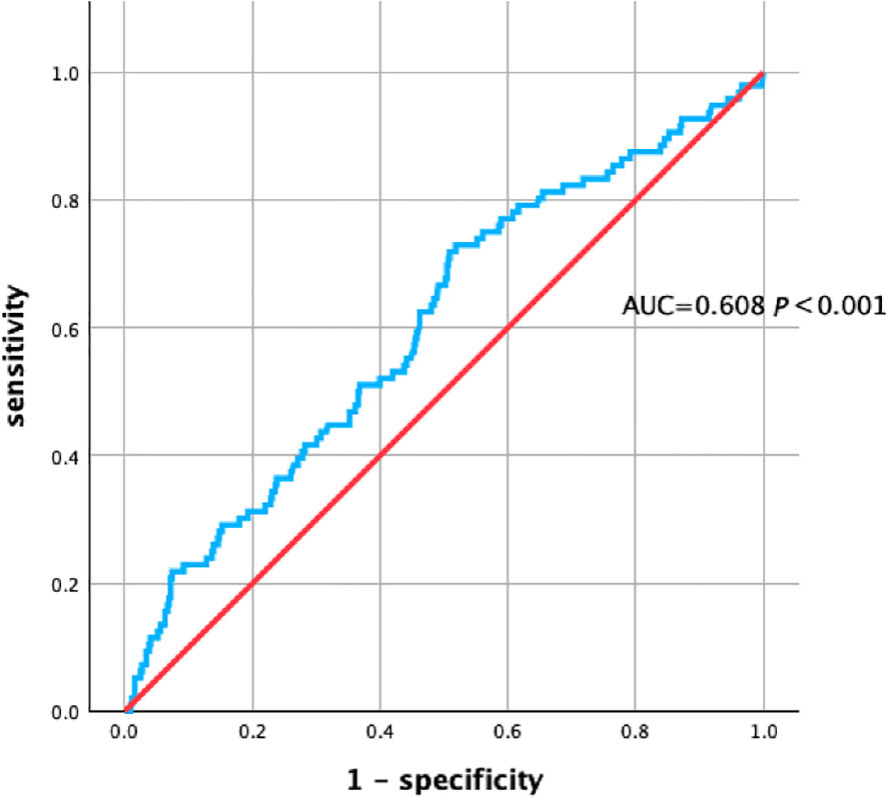
AUC of TG/HDL-C for the accuracy of prediction of the risk of PPCM. Peripartum cardiomyopathy, PPCM; triglyceride-to-high-density lipoprotein cholesterol ratio, TG/HDL-C; area under the curve, AUC.

According to the ROC curve analysis, the optimal cutoff value was 1.42. The area under the ROC curve for the prediction of PPCM onset using the TG/HDL-C ratio was 0.608 (95% CI, 0.545–0.671, *p* < 0.001), suggesting that the TG/HDL-C ratio has a certain level of predictive accuracy for the onset of PPCM (**Figure 3**).

According to the analysis of the correlation of the metabolic indicator (the TG/HDL-C ratio) with LVEDd, LVEF, and NT-proBNP, we found that the TG/HDL-C ratio was associated with NT-proBNP (**Table 5**). Therefore, we concluded that the TG/HDL-C ratio was correlated with the severity of HF at the onset of PPCM.

**Table 5:** Correlation analysis between TG/HDL-C and heart failure characteristic index.

		*r*	*p*
TG/HDL-C	NT-proBNP	0.360	0.001
	LVEDd	−0.123	0.246
	LVEF	0.110	0.286

TG/HDL-C, Triglyceride-to-high-density lipoprotein cholesterol ratio; NT-proBNP, N-terminal B-type natriuretic peptide; LVEDd, left ventricular end diastolic diameter; LVEF, left ventricular ejection fraction.

## 4 Discussion

In the present study, we found that the TG/HDL-C ratio was significantly higher in women with PPCM than in healthy controls, serving as an important predictor of PPCM occurrence. The ROC analysis revealed that the TG/HDL-C ratio had a certain predictive accuracy. To the best of our knowledge, this study is the first to explore the predictive value of the TG/HDL-C ratio in the development of PPCM in women during pregnancy and childbirth.

PPCM is a specific type of dilated cardiomyopathy that severely threatens maternal health and quality of life. Previous studies have revealed multiple births, a family history of cardiomyopathy, ethnicity, smoking, gestational hypertension, preeclampsia, diabetes, malnutrition, older age, and long-term use of tocolytic agents as the recognized risk factors for PPCM (5). In our study, there were no differences in age or BMI between the two groups, partly because of the small sample size and the selection bias introduced by sampling. Blood lipid tests are not routinely performed in obstetrics. The inclusion criterion was the presence of blood lipid tests upon admission, which may have led to selection bias. This may explain the absence of age differences between the two groups. Patients with PPCM had a higher rate of multiple pregnancies, higher blood pressure, and a higher probability of combined sinus arrhythmia. This is consistent with the results of previous studies. Multiple pregnancies and births are risk factors for PPCM. Moreover, multiple pregnancies and a prolific history will increase the physical burden on pregnant women, especially on the cardiovascular and metabolic systems. Simultaneously, multiple pregnancies and a prolific history will also increase the risk of pregnancy-related complications, such as pregnancy-induced hypertension and preeclampsia, which will further affect the cardiovascular system and increase the risk of PPCM (15).

Dyslipidemia is an important risk factor for cardiovascular diseases, and the LDL-C level is the most important risk factor for coronary atherosclerotic heart disease, the cause of which is unclear. No studies have explored the relationship between blood lipid levels and the incidence of PPCM. This study is the first to explore the relationship between blood lipid levels and the pathogenesis of perinatal cardiomyopathy. In this study, a comparison of the lipid levels and metabolic indices between the patients with PPCM and healthy perinatal pregnant women showed that there were significant differences in the TG/HDL-C ratio between these groups, which provides important clues for evaluating the susceptibility to PPCM. Patients with PPCM had higher uric acid levels than controls, suggesting metabolic abnormalities. Previous studies have found metabolic abnormalities, including lipid metabolism (16), in induced pluripotent stem cell-derived cardiomyocytes in patients with PPCM (17), and key metabolic gene abnormalities were found in the STAT3-CKO mouse model, which is consistent with the conclusion reached in previous basic trials that metabolic regulation is a key factor in the susceptibility to PPCM and abnormal metabolic regulation increases susceptibility to PPCM (17). Under normal physiological conditions, maternal lipid metabolism increases during the last trimester of pregnancy and quickly returns to normal levels after delivery. This metabolic transformation largely depends on transcription factors that control lipid metabolism. The results revealed that the regulation of lipid metabolism-related pathways in the patients with PPCM was disrupted, lipid metabolism was inhibited, and anaerobic glycolysis was impaired (17). Aging individuals rely mainly on glycolysis to produce energy for glucose utilization, which may promote the development of perinatal cardiomyopathy. In this study, we used novel metabolic indicators to better reflect the state of metabolism in the body of patients with PPCM and found significant differences between the TG/HDL-C ratio in patients with PPCM and healthy perinatal women. Building upon prior studies, we found that a higher TG/HDL-C ratio increased maternal susceptibility to PPCM. This may be related to a lipid metabolism disorder, which affects the utilization of glucose by myocardial cells and promotes the occurrence of HF.

NT-proBNP is a hormone produced by the heart that is released in response to stress and an increased load on the cardiovascular system (18). The release of NT-proBNP is closely associated with an increase in ventricular pressure and tension. Its main function is to alleviate cardiac load by promoting fluid excretion and vasodilation (18). Therefore, when cardiac wall tension increases, NT-proBNP levels may also increase, which can be considered a physiological response. Clinically, by measuring the NT-proBNP levels, we can assess the severity of HF and predict cardiovascular risk. NT-proBNP is the only clinically valuable diagnostic biomarker usually used in PPCM and has prognostic value in predicting the recovery of the left ventricle in patients with PPCM (19). In this study, we found that the TG/HDL-C ratio was associated with the NT-proBNP levels. Therefore, we concluded that the TG/HDL-C ratio was correlated with the severity of HF at the onset of PPCM. Although the TG/HDL-C ratio may be associated with the prognosis of patients with PPCM, further follow-up studies are needed to confirm this.

Patients with PPCM had lower albumin levels than healthy controls. Hypoalbuminemia is a risk factor for PPCM, which is consistent with the findings of previous studies. However, epidemiological studies on thromboembolism in patients with PPCM have not been widely conducted. Various studies have reported thromboembolic incidence risk rates ranging from 3% to 16% in patients with PPCM, which are higher than those in the general population (20). Elevated hormone levels during pregnancy can lead to hypercoagulability. In patients with PPCM, decreased left ventricular contractility further contributes to blood stasis, increasing the risk of thrombosis. In our study, the patients with PPCM had prolonged clotting time *in vivo* and lower fibrinogen levels. Moreover, the incidence of thromboembolic events was significantly increased in 150 patients with PPCM. Among them, approximately 14.7% of the patients had intracardiac thrombi, one had pulmonary embolism, and one had lower-limb venous thrombosis. The incidence of thrombosis was higher in the PPCM group; however, the PPCM group had a prolonged clotting time and decreased fibrinogen levels, which may be related to a selection bias. Further clinical research with larger sample sizes is required to confirm these findings.

Patients with PPCM usually exhibit decreased myocardial contractility, insufficient liver and kidney perfusion, and varying degrees of damage to the liver and kidney function. In our study, compared to healthy women in the control group, patients with PPCM had higher transaminase levels and lower eGFR; however, most were within the normal range, which may be related to mild hypoperfusion of the liver and kidney in the acute stage of HF, with slight liver and renal function damage.

In our study, the patients with PPCM had abnormal metabolic indices, which may have increased their susceptibility to PPCM. This study is the first to explore the correlation between the TG/HDL-C ratio generated based on routine prenatal examinations and the incidence of PPCM, which has good economic benefits and can help alleviate the economic pressure on pregnant women, especially in underdeveloped areas. Other possible biomarkers for cardiomyopathy can also be analyzed in this population sample, as patients with PPCM showed alterations in their blood lipid and cell count values. We have analyzed the correlation between other possible composite biomarkers and patients with PPCM in this population sample, and that study is being reported. These indices are simple and easy to obtain, and the cost is low; however, their correlation with the onset of perinatal cardiomyopathy still needs to be confirmed by multicenter and large-sample research.

### 4.1 Limitations

This study had some limitations. First, because of the low incidence of PPCM, the number of PPCM cases included in this study was limited. This was a retrospective case–control study, and the data of patients with PPCM were obtained mainly through the hospital medical record system and telephone follow-ups, with recall bias. In addition, only 450 healthy women who gave birth at Qilu Hospital between January 2010 and August 2023 were randomly selected as the control group. This might have reduced the testing efficiency and resulted in a selection bias. Furthermore, most of the healthy mothers lacked echocardiography results, and the determination of PPCM was mainly via telephone follow-ups based on the absence of obvious chest tightness, dyspnea, edema, and other clinical manifestations in the third trimester, as well as no obvious clinical manifestations in the postpartum months. As there was no echocardiography to prove that the control group did not have PPCM, some asymptomatic patients may have been included in the control group. Finally, the relationship between the onset of PPCM and the levels of inflammatory biomarkers upon admission needs to be clarified in larger prospective studies.

## 5 Conclusions

A higher TG/HDL-C ratio is significantly associated with the risk of PPCM. The TG/HDL-C ratio was positively correlated with the NT-proBNP levels at the onset of PPCM.

## Data Availability

The raw data supporting the conclusions of this article will be made available by the authors, without undue reservation.
